# A Book Interaction Scheme to Enhance Children’s Reading Experiences and Preferences

**DOI:** 10.3389/fpsyg.2020.02155

**Published:** 2020-10-14

**Authors:** Mingming Zhang, Guanhua Hou, Yeh-Cheng Chen, Tao Zhang, Jie Yang

**Affiliations:** ^1^School of Design, Jiangnan University, Wuxi, China; ^2^Pan Tianshou College of Architecture, Art and Design, Ningbo University, Ningbo, China; ^3^Department of Computer Science, University of California, Davis, Davis, CA, United States; ^4^School of Artificial Intelligence and Computer Science, Jiangnan University, Wuxi, China; ^5^Institute of Image Processing and Pattern Recognition, Shanghai Jiao Tong University, Shanghai, China

**Keywords:** interaction design, user experience, usability, affective experience, reading preference

## Abstract

The interaction between children and books is an essential part of the reading experience. Publishers all over the world are working to cultivate reading habits in children and attract attention to traditional books. Considering the invaluable nature of these early reading experiences. This paper investigated the effects of book interaction design on 5–6 years old children, taking into account reading preferences, measuring reading time, and emotional response to improve their reading experience and potentially design books according to these interactions. The results showed that preschool children (5–6 years old) prefer sensory interaction, and that book interaction design has a significant influence on reading time, affective experience, and subjective ratings. Girls around 5–6 years preferred folding interaction and pop-up interaction in reading. This study summarizes these results in order to provide practical guidelines for book publishers, enabling them to design better books for children.

## Introduction

Book interaction scheme refers to the interaction style between human and book, which enable promising applications to meet the individual needs of readers, creating company business models and integrating smart technologies as part of book development. Interaction with and emotional responses to books are important for younger children’s reading, with repercussions on book selecting, reading pleasure, and reading strategy. It is thus important to consider the interaction accessibility and emotional experience of books for younger children, and how this affects their decisions when choosing books, as younger children may struggle or switch their attention to other activities. This is especially important when we consider that in recent years, reading for pleasure has declined, especially among children (National Center for Education Statistics, 2001).

Children-book interaction design describes an interactive communication design process that takes into account the relationship children have with books and their reading processes. The interactive content includes interaction and operability. By interacting with books, children’s visual, auditory, tactile, and other cognitive abilities are enhanced. Interaction increases children’s interest and creativity in reading. There are two types of children’s book interaction methods: behavioral interaction and sensory interaction. Behavioral interactions include page flipping, pop-up interaction, draw interaction, rotation interaction, and folding interaction, among others. Sensory interaction includes visual interaction, audio interaction, tactile interaction, and olfactory interaction (Littleton et al., 2006). Audio interaction helps to improve children’s phonological awareness and change reading strategies (Chera and Wood, 2003; Wood, 2010). In this study, we focused on the effects of tactile and olfactory interaction on children’s reading preferences.

Reading preferences and interests evolve with age, for example, young children often prefer fictional stories that use the imagination, and older children are often interested in more realistic fiction (Fisher, 1988; Boraks et al., 1997). There were lots of studies focused on older children in middle or high school, but there is little research on younger children, even though early experiences of reading help to develop reading habits in later life (Cetin and Bay, 2014). Younger children in kindergarten/nursery are easily encouraged to interact with books, for example, they are attracted to the aesthetics pictures on the covers or in the contents (Fast, 2000). Therefore, this study focused on 5–6-year-old preschool children (preschool children for short) and investigated the effects of book interactions on their reading experience.

Compared with digital reading, traditional books help protect eyesight and are less dependent on computers (Jeong, 2012; Hou et al., 2020). More people have in recent years become addicted to digital reading, whether adolescents (Lee et al., 2014) or adults (Elhai et al., 2016). It is irresponsible to ask preschool children to do lots of digital reading in kindergarten, early education can be used as an opportunity to develop good reading habits and attract them to books. This study aimed to investigate the effects of interaction with books on the reading preferences of younger children, in developing educational guidelines for developing reading habits in preschool children.

Gender is another important factor that needs to be considered in early reading. It affects a child’s preference for shapes, colors, and toys, with significant gender differences during infancy (Jadva et al., 2010; Hou and Lu, 2019). As they grow older, this gender difference becomes more obvious is often formed around gender-stereotypes, for example, the idea that boys prefer toys with so-called “male characteristics” such as cars and weapons or that girls prefer dolls and to “play house.” The theory of social learning points out that gender differences arise from the ways in which male and female social roles are portrayed and represented through culture (Auyeung et al., 2009). In addition, studies on these culturally conditioned differences in terms of morphological preference have found that girls prefer flowers, butterflies, and figures, while boys prefer items such as cars, trains, and rockets (Langerman, 1990; Lytton and Romney, 1991). These gender stereotypes inform differences and interest in reading content. Previous studies have indicated that the preferences of girls and boys are different when selecting a book, for example, surveys of first-grade students in the elementary schools in Ohio, suggested that girls overwhelmingly prefer narrative texts while boys prefer nonfiction (Harkrader and Moore, 1997). These gender differences are significant when related to the social aspects of recreational reading, and perceived reading ability (Mohr, 2006). Thus, we hypothesize that gender may have a significant effect on interaction preferences.

Taken together, the reading experience is a complex process. Previous studies have used surveys or questionnaires rather than actual observation of children’s reading interaction behavior. Many researchers have focused on a single aspect of books, such as aesthetics (the appearance of a book cover), the presence of illustrations, or the book’s selecting. This study investigated book interaction and gender together and addressed the following research questions:

1. What kind of emotional experience do children expect when reading a book?
2. Which kind of interaction do younger children prefer, behavioral interaction or sensory interaction?
3. Is the effect of book interaction design significant for children’s reading time, reading preferences, and emotional experience?
4. Is gender significant in reading time, interaction preferences, and emotional experience?

## Materials and Methods

The study involving human participants and was reviewed and approved by the Design Lab of Jiangnan University.

### Materials

Six of the most common interaction styles in children’s books were selected as experimental material, including behavioral interaction and sensory interaction (Detemple and Tabors, 1994). These are flip interaction, draw interaction, pop-up interaction, folding interaction, tactile interaction, and olfactory interaction. Page flip interaction means scrolling from left to right or right to left. Draw interaction means changing the content of the picture by pulling or drawing. Pop-up interaction means that the picture in the book will pop up when it is opened, and the stereo image will increase the sense of space. Folding interaction refers to changing the picture by folding to form a new reading content. Tactile interaction means knowing by touching, for example, there might be plastic, sand, or other materials in the book. Olfactory interaction refers to the addition of flavors associated with the content, such as flowers.

**FIGURE 1:**
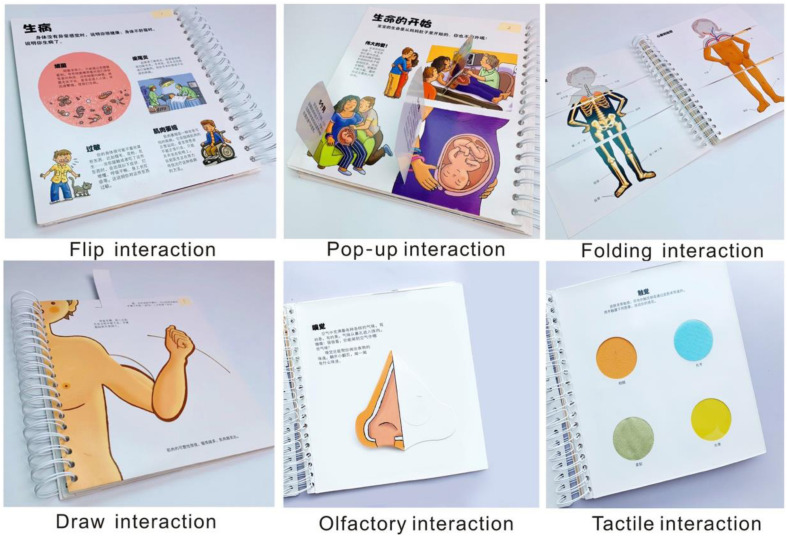
Experimental materials.

In order to exclude the influence of the content of the reading materials on participants, this study designed six children’s books on the theme of human body structure. The reading materials are similar, and each reading material contains only one interactive form. Experimental materials are shown in Figure 1.

### Materials Measurement and Emotional Reaction

**FIGURE 2:**
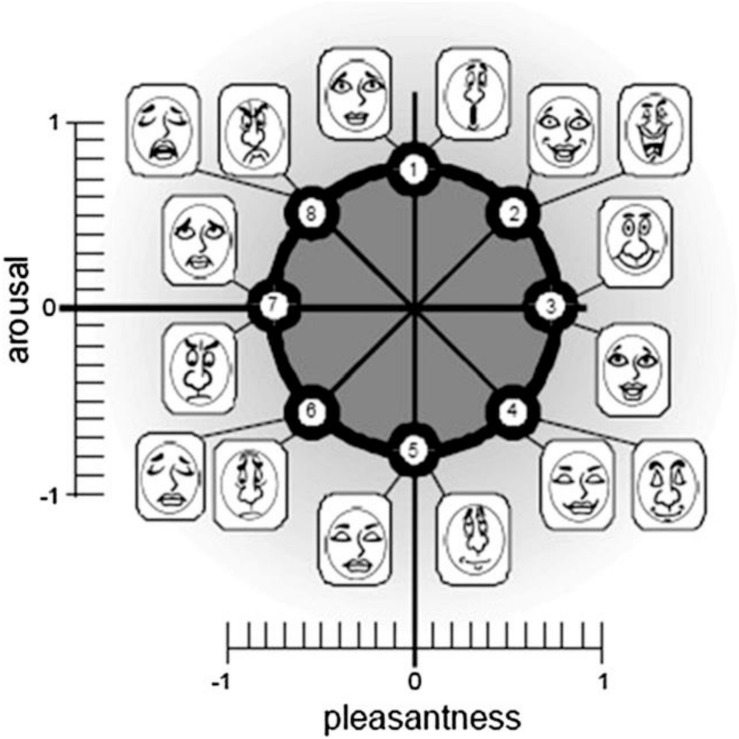
16 Russell’s emotion card.

The perception of each child participant was subjective and intuitive, with their emotions directly reflecting their preferences. Although there are many methods of measuring emotion, such as the self-report, autonomic nervous system measurement, startle response measurement, behavioral measurement, and brain measurement, there are few suitable methods of measuring these emotions in children. The most common methods of measuring emotions in children usually involve self-reports and behavioral measurements. Self-reports include the positive-negative emotion scale (PNANS), the pleasure, arousal, and dominance scale (PAD), and Russell’s circular emotional card (Russell, 1980; Desmet et al., 2001; Cheng et al., 2018). Behavioral measurements include facial expression recognition and volume changes, but behavioral measurements require a longer experiment time and are therefore not suitable for child participants. Russell’s emotion card divides emotion into 8 types according to the PAD scale, measuring whether they enjoyed the experience and how much the reading experience engaged their interest. Each mood consists of 2 expression pictures and a total of 16 cards (Russell, 1980), as shown in Figure 2. The scale is intuitive, easy to read, and easy to identify. This method was more suitable for child participants as they only need to select one of the cards to express their feelings. Before the experiment began, we asked each child participant to read the emotional card and ensured that they understood each emotion in the picture and could confidently choose the picture that best reflected their mood.

### Experimental Design

This study used a two-factor experimental design within and between groups. Book interaction style and child gender were two independent variables. The book interactions included page flip interaction, pop-up interaction, rotation interaction, touch, and olfactory interaction, pull interaction, and folding interaction. Interaction style was a within-group factor, while gender was a between-group factor. Reading sequences were arranged according to the Latin Square Sequence.

Children’s behavior and reading time were recorded by a camera. After each session, the participants completed the following tasks: firstly, they chose an emotional card to match their emotions; then secondly, they used 5 sticky stars to rate the reading materials.

### Participants

The experiment was conducted in a kindergarten library for 2 weeks. A total of 40 kindergarten classmates were invited to participate in the experiment. The age of participants was around 5–6 years old, including 20 boys and 20 girls. Each student had a study time of more than 2 h per week in the kindergarten library and extensive previous library reading experience, to ensure the smooth progress of this experiment. All children participating in the experiment had normal vision. Before the experiment, the teachers authorized each child’s participation and carefully read the informed consent form, which confirmed that the study would cause no physical or psychological harm to the children.

According to the experimental design plan, the children were divided into two groups, male and female, with 20 people in each group. They read the reading materials according to a pre-set reading order.

### Tasks and Data Collection

After the children entered the library, they listened to an introduction to the experiment, which outlined its content and its requirements. The researchers involved with his study taught participants how to read and distinguish Russell’s emotional card of 16 pictures to ensure that participants understood these emotions. They were then asked to select the emotional experience they expected to have from reading. After that, the experiment started and participants read the experimental materials in turn. After they had finished, they chose a card to match their mood. The participants used the sticky stars to rate their preferred reading materials, with five stars indicating that they liked it very much, and one star representing that they did not like it at all.

Data from a total of 4 groups data was collected, including (1) each child’s expected emotional experience from reading;(2) reading time; (3) each child’s emotional experience after reading each material; and (4) the star ratings for each material. Each child had an unrestricted reading time, and the duration of reading began when they started reading and stopped when they were tired of reading. Data were analyzed by SPSS 19.0.

## Results

### Expected Emotions and After Task Emotions

#### Expected Emotional Experience When Reading

**FIGURE 3:**
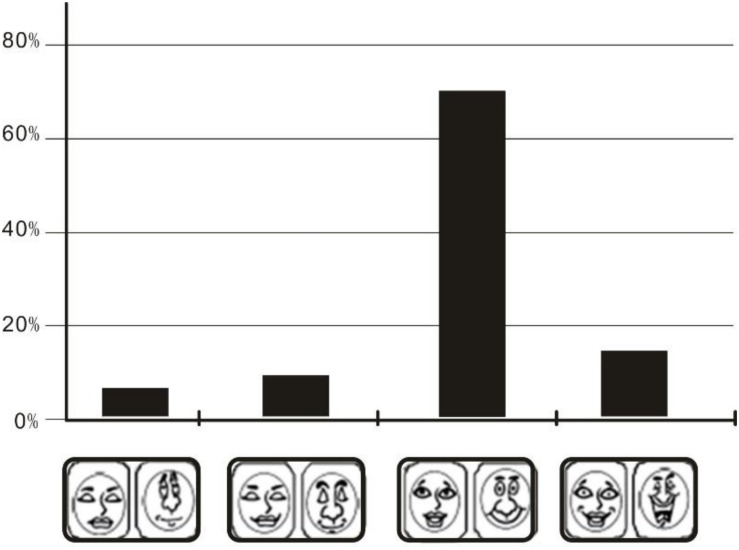
Emotional ring card emotional distribution.

The expected emotional experience in reading, as measured by Russell’s emotional card before the experiment, revealed that most children expected to have a pleasurable emotional experience with a slight increase in their interest (based on the PAD scale), as shown in Figure 3. Over 70% of the children expected to have an enjoyable, positive reading experience. Approximately 15% of the children expected reading to be a highly engaging experience.

#### After Tasks’ Emotional Experience in Reading

**FIGURE 4:**
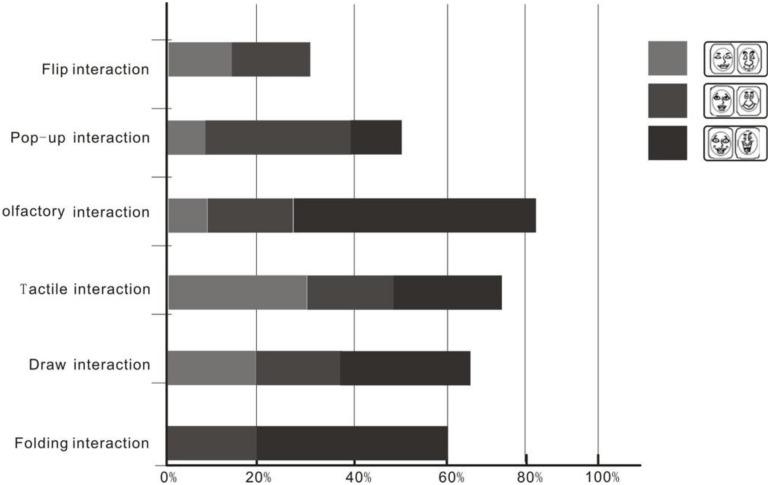
Proportional distribution of positive emotions in participants by interaction style.

After reading the experimental materials, participants were asked to indicate the card that represented their emotional feeling. The emotions were induced by interaction style, including positive emotions (happiness or pleasure), and negative emotions (anger and sadness). This study focused on the positive emotions induced by book interactions. Over 80% of participants had a positive emotional experience by olfactory interaction, followed by tactile olfaction interaction, and 75% of participants perceived reading as a positive emotional experience. The page flip interaction induced the lowest positive emotional experience, which only accounts for 35% of participants. The results are shown in Figure 4. The color darkness in the figure represents the level of interest, and the black refers to a higher interest.

Compared with the expected emotional reading experience, the emotional experience induced by olfactory interaction was the most in line with the expected emotional experience. Followed by tactile and folding interaction.

#### Gender and Perceived Emotional Experience

A chi-square test of statistical results revealed that the positive emotional influence of the book interaction patterns induced significantly different responses (χ^2^ = 6.93, *p* < 0.01). In addition, the results showed that gender had significance (χ^2^ = 4.19, *p* = 0.031), with girls are more affected by pop-up and folding interactions than boys.

### Reading Time, Subjective Rating, and Interaction Preferences

#### Gender Effects and Emotional Experience

The time it took for participants to read each experimental material is summarized in Table 1. These results indicate that participants spent the shortest amount of time on page flip interaction, with a mean reading time of about 21.05 s, while they spent longer on folding interaction, with a mean time of 66.35 s. This was followed by olfactory interaction, which had a mean of 58.1 s.

**TABLE 1:** Distribution of child reading time and different book interaction modes.

**Interactions**	***N***	**Mean**	**Standard deviation**	**Standard error**	**95% confidence interval for the mean**		**Minimum**	**Maximum**
					**Lower limit**	**Upper limit**		
Page flip	40	21.0500	12.89012	2.88232	15.0172	27.0828	6.00	49.00
Pop-up	40	27.2500	12.85087	2.87354	21.2356	33.2644	4.00	51.00
olfactory	40	58.1000	31.20880	6.97850	43.4938	72.7062	10.00	148.00
Tactile	40	40.3500	19.40503	4.33910	31.2682	49.4318	20.00	99.00
Draw	40	35.6000	11.11377	2.48511	30.3986	40.8014	11.00	59.00
Folding	40	66.3500	30.70621	6.86612	51.9791	80.7209	27.00	142.00

Two-way analysis of variance was used to analyze reading time. Results showed that the level of interaction with a book had a significant impact on reading time (*F* = 13.54, *p* < 0.01, $\eta_{\text{p}}^{2}$ = 0.76). Gender had no significant difference in reading time (*F* = 1.14, *p* = 0.287, $\eta_{\text{p}}^{2}$ = 0.13). We compared the reading time of each of the six interactions, and the difference between page flip interaction and pop-up interaction was not significant (*F* = 1.08, *p* = 0.362, $\eta_{\text{p}}^{2}$ = 0.08). Mean reading time in flipped interactive books is significantly lower than that of olfactory (*F* = 9.91, *p* < 0.01,$\eta_{\text{p}}^{2}$ = 0.54), tactile (*F* = 7.57, *p* = 0.011, $\eta_{\text{p}}^{2}$ = 0.47), draw (*F* = 6.32, *p* = 0.025,$\eta_{\text{p}}^{2}$ = 0.32), and folding interaction (*F* = 11.25, *p* < 0.01, $\eta_{\text{p}}^{2}$ = 0.63). The mean reading time spent on pop-up interactive books is significantly lower than interaction with olfactory (*F* = 10.32, *p* < 0.01, $\eta_{\text{p}}^{2}$ = 0.59) and folding (*F* = 7.83, *p* < 0.01, $\eta_{\text{p}}^{2}$ = 0.51). The mean reading time of olfactory interaction books was significantly longer than tactile (*F* = 5.89, *p* = 0.038, $\eta_{\text{p}}^{2}$ = 0.29) and draw interaction (*F* = 9.33, *p* < 0.01, $\eta_{\text{p}}^{2}$ = 0.91), and the difference between olfactory interaction and folding interaction is not significant (*F* = 0.98, *p* = 0.825, $\eta_{\text{p}}^{2}$ = 0.01). Participants’ mean reading time for tactile interaction books was not significantly different from that of draw interaction books (*F* = 0.77, *p* = 0.484, $\eta_{\text{p}}^{2}$ = 0.11), but it was significantly lower than the reading time in folding interaction books (*F* = 8.32, *p* < 0.01, $\eta_{\text{p}}^{2}$ = 0.82).

#### Gender Effect on Perceived Emotional Experience

Once the reading task was completed, participants used the five-pointed stars to evaluate their interaction, with rating scores shown in Table 2. The highest mean score was obtained by olfactory interaction(M = 4.55, SD = 0.82), followed by folding interaction (M = 3.85, SD = 1.30), draw interaction (M = 3.88, SD = 1.36), tactile interaction (M = 3.35, SD = 1.13), pop-up interaction (M = 3.15, SD = 1.34), and flip interaction (M = 1.70, SD = 1.21). The mean rating score of olfactory interaction was the highest, and page flip interaction was the lowest.

**TABLE 2:** Participants’ scores for interaction with reading materials.

**Interactions**	***N***	**Mean**	**Standard deviation**	**Standard error**	**95% confidence interval for the mean**		**Minimum**	**Maximum**
					**Lower limit**	**Upper limit**		
Flip	40	1.7000	1.21828	0.27242	1.1298	2.2702	0.00	4.00
Pop-up	40	3.1500	1.34849	0.30153	2.5189	3.7811	1.00	5.00
olfactory	40	4.5500	0.82558	0.18460	4.1636	4.9364	2.00	5.00
Tactile	40	3.3500	1.13671	0.25418	2.8180	3.8820	1.00	5.00
Draw	40	3.8000	1.36111	0.30435	3.1630	4.4370	1.00	5.00
Folding	40	3.8500	1.30888	0.29267	3.2374	4.4626	1.00	5.00

Statistical analysis of the above scores found that the effect of book interaction on children’s subjective evaluation was significant (*F* = 12.59, *p* < 0.01, $\eta_{\text{p}}^{2}$ = 0.71). The effect of gender on evaluation was not significant (*F* = 0.363, *p* = 0.548, $\eta_{\text{p}}^{2}$ = 0.03). A comparison of the subjective scores of the six interaction methods revealed that the flip interaction was lower than the other five interaction methods and that it was the most unpopular method of interacting with books. The subjective evaluation of the pop-up interaction was significantly lower than olfactory interaction (*F* = 5.58, *p* < 0.01, $\eta_{\text{p}}^{2}$ = 0.41), but the differences between pop-up interaction and tactile, drawing, or folding interaction were not significant. The subjective evaluation of olfactory interaction was significantly higher than that of flip (*F* = 14.17, *p* < 0.01, $\eta_{\text{p}}^{2}$ = 0.77), pop-up (*F* = 8.42, *p* < 0.01, $\eta_{\text{p}}^{2}$ = 0.62), and tactile interaction (*F* = 6.77, *p* < 0.01, $\eta_{\text{p}}^{2}$ = 0.47), but the difference between drawing (*F* = 3.34, *p* = 0.053, $\eta_{\text{p}}^{2}$ = 0.24) and folding interaction (*F* = 2.81, *p* = 0.071, $\eta_{\text{p}}^{2}$ = 0.19) was not significant.

#### The Influence of Gender on Emotional Experience

The results showed that the most popular way for children to interact with books is sensory interaction, on which they dwell for a longer time [M(sensory) = 49.225 VS. M (behavioral) = 37.25], and was rated higher [M(sensory) = 3.95 VS. M(behavioral) = 3.13]. One-way ANOVA was used to analyze the difference between sensory interaction and behavioral interaction on reading time and subjective rating, and results showed that the time spent on sensory interaction was significantly longer than behavioral interaction (*F* = 7.13, *p* = 0.01, $\eta_{\text{p}}^{2}$ = 0.58). The rating for sensory interaction was significantly higher than behavioral interaction (*F* = 4.81, *p* = 0.043, $\eta_{\text{p}}^{2}$ = 0.36).

A gender difference analysis was performed on the ratings, and it was found that female children had a significant preference for pop-ups (*F* = 5.217, *p* = 0.032, $\eta_{\text{p}}^{2}$ = 0.39) and folding interactions (*F* = 3.891, *p* = 0.050, $\eta_{\text{p}}^{2}$ = 0.28) compared to male participants. It may indicate that gender influences a child’s preferred interaction style.

## Discussion

Reading is an important part of life and it is crucial to ensure that young children developing good reading habits in preschool so that they can obtain knowledge, enhance their interests in learning, and cultivate life-long reading abilities. Children’s choices when selecting books are not based on professional and academic standards and are instead dictated by vividness and liveliness, as they increase their interest (Fast, 2000; Cetin and Bay, 2014; Guo et al., 2015). The interactive form of books plays an important role in attracting interest. Thus, this study analyzed the influence of book interactions on reading time, emotional experience, and preference, and summarized these conclusions as suggestions for encouraging preschool children to read.

### Resource Identification Initiative

Children expect a pleasant emotional experience when reading a book, which could be derived from story content, illustrations, and their surroundings. Preschoolers like to read colorful and beautiful pictures (Hargrave, 2001; Justice and Lankford, 2002) and listen to stories (Anvari et al., 2002). This study found that they do not only experience pleasure from the story, but that their enjoyment is also connected to their interaction and experience when reading books. This study found that olfactory interaction, tactile interaction, and folding interaction can induce positive emotions in line with their expectations. Hargrave (2001) found that preschoolers pay more attention to the form of books than to content. When new forms of interaction are combined with content, they can induce pleasure in preschool children. Furthermore, Leech and Rowe (2014) found that picture books can be easily understood by preschool children and can lead to subsequent discussions about this content with their parents, meaning that they also help children’s language development. Preschoolers can easily interact with books according to their intentions, which increases their sense of participation and enhances positive emotions.

These positive emotions could be induced by different cognitive mechanisms, and contribute to enhancing their motivation to read (Seo et al., 2010). The positive emotions induced by listening stories are different from those that are induced by a change of the book’s method of interaction. For example, when children participate in listening, understanding, and imagining, they experience emotional changes that result from their immersion and the consistency of their expectations about the ending of the story (Mohr, 2006; Wei et al., 2018). However, the book’s interaction induces a positive emotion, based on cognitive processes, such as visual cognition, creative thinking, and operational execution. Book interaction is an important means of improving the reading experience of children, as they are likely to feel a positive emotion once their expectations are fulfilled, and will be encouraged to read again in the future.

### Reading Interests and Preference

Reading interests and preferences evolve with age. Attracting preschoolers to reading and encouraging them to spontaneously engage in it, is crucial. This study evaluated children’s interest in reading by examining reading time. A longer reading time often indicates a greater interest (Hou and Lu, 2018). We also explored the effect of book interaction on reading time and the reading preferences of preschool children. Results showed that book interaction has a significant impact on reading interest, suggesting there are significant differences in the reading time associated with different interactive reading materials. For preschool children, the most attractive interactive book form was folding interaction, while the overall sensory interaction was higher than behavioral interaction. Preschool children are curious, they read pictures rather than words, and pictures helped them understand the story (Leech and Rowe, 2014; Li and Zhu, 2017), thus interactions that changed the picture into a different story attracted them the most. Behavioral interaction is an important factor in the design of books and could affect how they display illustrations and words. The results of this study show that interaction design is a good way to encourage preschool children to read.

The rating scores attributed by preschool children to book interactions resulted in a higher mean score for tactile and olfactory interactions compared with behavioral interactions. The differences between olfactory interaction, folding interaction, and draw interaction were not significant, indicating that preschool children preferred these designs to pop-up, tactile, and flip interaction. The mean value of olfactory interaction scores was the highest because this type of interaction is rare in everyday books, and reading a book by smell went beyond the expectations of the participants. Folding interaction is related to the presentation of book content, and changes the content of books and enhances the novelty of materials, as the story continued by unfolding the book. The drawing-based interaction is also easy to access, and the content and form are well-matched, and the score was also high. The analysis found that subjective scores positively correlated with reading time (*r* = 0.74, *p* = 0.012). The longer the reading time, the higher the score for the corresponding book interaction style. In summary, the interactive form of books has a significant impact on the reading interest, subjective ratings, and preferences of preschool children. This indicates that a reasonable way to interact with books helps to improve the quality of a child’s reading experience.

### Gender Effects

Gender differences often affect all aspects of life. When it comes to reading, gender differences weres reflected in the different choices of color, content, and forms of the books chosen by participants (Jadva et al., 2010). This study found that the gender difference in reading time is not significant, and boys and girls spent a similar amount of time reading the same book for each of the different interaction styles. However, the gender difference was significant in terms of participant preferences. This study found that girls prefer pop-up interaction and folding interactions, while boys rated these types of interaction much lower. In other forms of interaction, the ratings of book forms were similar. The participant reading interests were not affected by gender, but subjective ratings of book interactions did have significant differences.

Gender differences were also reflected in the content of the books read, as well as in the participants’ selection of particular books, which may already be informed by their reading habits (Fisher, 1988; Boraks et al., 1997). Even though gender differences do exist in society, this factor is not widely considered in the scope of early education. Considering the influence of gender in book interaction design is beneficial to improve the quality of children’s reading experience.

### Implications and Design Recommendations

Despite the fact that flip interaction is the most frequent interaction in children’s books, it was not fascinating for preschool children. Based on the results of this study, suggestions about which book interaction formats might be most effective in designing children’s books are summarized in Table 3. Firstly, olfactory interaction, tactile interaction, and folding interaction are excellent choices, because these interactions could induce more positive emotional experiences and get higher subjective rating scores in preschool children. Tactile and olfactory interactions are seldom used in children’s books. Secondly, it is important to consider gender effect and how it informs children’s book selection. According to the results, tactile olfactory interaction should be considered a priority for both boys and girls, but folding interaction and pop-up interaction could potentially incite positive reactions in girls. Teachers should make full use of these interactions as a way of encouraging reading habits in preschool children. This study examined the effects of each interaction on children’s emotional experience, reading time, ratings, and preference, to improve the overall reading experience for children and help teachers to design more appropriate activities for preschool children.

**TABLE 3:** Summary of early reading recommendations.

**Interactions**	**Recommendations for teachers**	**Gender priority**
olfactory	Use of olfactory interaction in children’s books can not only help children understand things, but also improve their reading interests and reading experience.	–
Tactile	Preschool children could know by touching, as it helps improve their reading experience.	–
Draw	Adding animation to a book helps improve the reading experience.	–
Folding	Girls like folding more than boys. Extending or changing stories aimed at girls, could improve the reading experience.	Girls
Pop-up	Girls pop up books more than boys, but their overall average preference was medium.	Girls
Flip	Could be used with other interactions.	–

### Limitations

Because emotional responses are inconsistent and complicated, there are limitations in the accuracy of the Russell’s emotional record card, used in this experiment. Although the target population is too young to use biometric measurements, future studies should consider other methods to ensure data is more objective in further studies. The age of participants was between 5 and 6 years old and due to dramatic changes in reading interests and preferences between different age groups, these conclusions are confined to this population. There are also more than 6 interaction styles in children’s book design, and we hope to analyze more interactions in future studies.

## Conclusion

Through experimental research methods, this study clarified the effects of book interaction methods and factors such as gender on preschool children’s reading interests, including their subjective evaluation, preferences, and emotional responses to reading. These results indicate that preschool children prefer sensory interactive reading materials. In addition, their overall evaluation of folding interaction and drawing-based interaction was also high. Preschool children look forward to having a pleasant emotional experience when reading, which can be satisfied by olfactory, tactile, and folding reading interactions. Gender differences are observed only in the interactive forms, namely folding and pop-up interactions, which were preferred by female children. These conclusions can help teachers better understand the expectations of preschool children, and provide a theoretical reference for early education.

## Data Availability Statement

The raw data supporting the conclusions of this article will be made available by the authors, without undue reservation.

## Ethics Statement

The studies involving human participants were reviewed and approved by Design Lab of Jinan Universtiy. Written informed consent to participate in this study was provided by the participants’ legal guardian/next of kin.

## Author Contributions

MZ wrote the manuscript. GH was in charge of experiment design. Y-CC dealed with data analysis. TZ was in charge of experiment organization. JY was in charge of big data and deep learning. All authors contributed to the article and approved the submitted version.

## Conflict of Interest

The authors declare that the research was conducted in the absence of any commercial or financial relationships that could be construed as a potential conflict of interest.
